# Effect of wet storage conditions on potato tuber transcriptome, phytohormones and growth

**DOI:** 10.1186/s12870-019-1875-y

**Published:** 2019-06-17

**Authors:** Bahram Peivastegan, Iman Hadizadeh, Johanna Nykyri, Kåre Lehmann Nielsen, Panu Somervuo, Nina Sipari, Cuong Tran, Minna Pirhonen

**Affiliations:** 1 Department of Agricultural Sciences, University of Helsinki, Helsinki, Finland; 2 Department of Chemistry and Bioscience, Aalborg University, Aalborg, Denmark; 3 Department of Biosciences, University of Helsinki, Helsinki, Finland; 4 Viikki Metabolomics Unit, Department of Biosciences, University of Helsinki, Helsinki, Finland; 5 Present address: Department of Biology, Lund University, Lund, Sweden

**Keywords:** Potato, Tuber, Storage, Water, Oxygen, Microarray, RNA-Seq, Growth, Defence, Energy metabolism

## Abstract

**Background:**

Stored potato (*Solanum tuberosum* L.) tubers are sensitive to wet conditions that can cause rotting in long-term storage. To study the effect of water on the tuber surface during storage, microarray analysis, RNA-Seq profiling, qRT-PCR and phytohormone measurements were performed to study gene expression and hormone content in wet tubers incubated at two temperatures: 4 °C and 15 °C. The growth of the plants was also observed in a greenhouse after the incubation of tubers in wet conditions.

**Results:**

Wet conditions induced a low-oxygen response, suggesting reduced oxygen availability in wet tubers at both temperatures when compared to that in the corresponding dry samples. Wet conditions induced genes coding for heat shock proteins, as well as proteins involved in fermentative energy production and defense against reactive oxygen species (ROS), which are transcripts that have been previously associated with low-oxygen stress in hypoxic or anoxic conditions. Wet treatment also induced senescence-related gene expression and genes involved in cell wall loosening, but downregulated genes encoding protease inhibitors and proteins involved in chloroplast functions and in the biosynthesis of secondary metabolites. Many genes involved in the production of phytohormones and signaling were also affected by wet conditions, suggesting altered regulation of growth by wet conditions. Hormone measurements after incubation showed increased salicylic acid (SA), abscisic acid (ABA) and auxin (IAA) concentrations as well as reduced production of jasmonate 12-oxo-phytodienoic acid (OPDA) in wet tubers. After incubation in wet conditions, the tubers produced fewer stems and more roots compared to controls incubated in dry conditions.

**Conclusions:**

In wet conditions, tubers invest in ROS protection and defense against the abiotic stress caused by reduced oxygen due to excessive water. Changes in ABA, SA and IAA that are antagonistic to jasmonates affect growth and defenses, causing induction of root growth and rendering tubers susceptible to necrotrophic pathogens. Water on the tuber surface may function as a signal for growth, similar to germination of seeds.

**Electronic supplementary material:**

The online version of this article (10.1186/s12870-019-1875-y) contains supplementary material, which is available to authorized users.

## Background

Potato (*Solanum tuberosum* L.) is the fourth most cultivated crop and the most important tuber-bearing plant worldwide, with production of approximately 380 million tons in 2016 [1]. Cultivated potato is auto-tetraploid (2n = 4x = 48) and highly heterozygous with an 850 Mb haploid genome that is 6 times larger than the *Arabidopsis thaliana* genome, making potato a challenging organism to study with molecular methods.

Potato tubers, similar to many fruits and vegetables, are often stored for several months before they reach the market for fresh consumption or are used for products by the food industry. During this postharvest period, tubers are exposed to both abiotic and biotic stresses. Insufficient ventilation in storage can cause increased temperature, leading to enhanced respiration of the tubers, which induces condensation that produces a film of water on the tuber surfaces. Water condensation can occur when the air temperature in storage is higher than the actual temperature of the tuber surface. The water film leads to a reduction in gas exchange between the tissues and air because the diffusion of oxygen in water is reduced 10^4^ times compared to that of air [2]. The effect of water on green plants from flooding or submergence in the field has been well characterized [3]. During flooding, low oxygen concentrations leading to hypoxia or anoxia in plant tissues cause a reduction in cellular energy charge, a decrease in cytoplasmic pH, the production of reactive oxygen species (ROS) and the accumulation of toxic end products from anaerobic respiration. The reduction in gas exchange is accompanied by a reduction or depletion of oxygen; an increase in CO2 and ethylene (ET) concentration inside the plant tissue; and changes in the hormonal regulation of growth in flooded plants [3].

Stored fruits and other organs have both structural and biochemical preformed barriers as constitutive defenses that are present as a first obstacle against pathogen attack. Wet conditions in storage have been shown to impair resistance mechanisms of tubers to pathogens, possibly due to the inhibition of cell wall lignification and suberization that protect the tubers from pathogen invasion [4]. It has been observed that anaerobic conditions combined with a water layer on the tuber surface cause rotting of the tuber tissue, most likely as a result of reduced plant defense and increased bacterial growth, whereas the incubation of dry tubers in anoxic conditions does not lead to rotting [4, 5]. These results suggest that water is a crucial factor that promotes rotting during storage. However, it seems that the ability of water to cause anoxic conditions by blocking oxygen diffusion is not the main cause of rotting because dry, anoxic tubers do not rot [5]. Insight into the underlying mechanism that causes vulnerability of potato tubers and other stored vegetables to pathogens in wet, low-oxygen storage conditions is needed to fully understand the tuber response.

Transcriptome profiling offers one way to characterize plant responses; however, there have been few transcriptome studies to unravel the response of plant products to abiotic stress factors during storage [6, 7]. The aim of this work was to utilize transcriptome profiling to understand how wet conditions affect potato gene expression to promote postharvest rotting. A microarray experiment was carried out with wet tubers in a low-oxygen conditions at a low storage temperature to characterize tuber response in these conditions. To mimic the unsuitable conditions at higher temperatures and to characterize the early response of tubers to water, an RNA-Seq study was conducted with three different time points, the earliest after 1 h expose to water. Quantitative real-time polymerase chain reaction (qRT-PCR), hormone measurements and plant cultivation were performed to verify the transcriptome results.

## Results and discussion

### Microarray and RNA-Seq profiling of potato tubers incubated in wet and dry conditions

To study the molecular processes promoting the rotting of wet potato tubers during long-term storage, transcriptomes of surface sterilized tubers incubated in wet and dry conditions were compared with microarrays and RNA-Seq in two separate experiments using tubers of cultivar (cv.) Bintje. To prevent the drying of the tubers during the experiment, all the tubers were kept in closed bags, which created low-oxygen conditions in all samples.

**Fig. 1:**
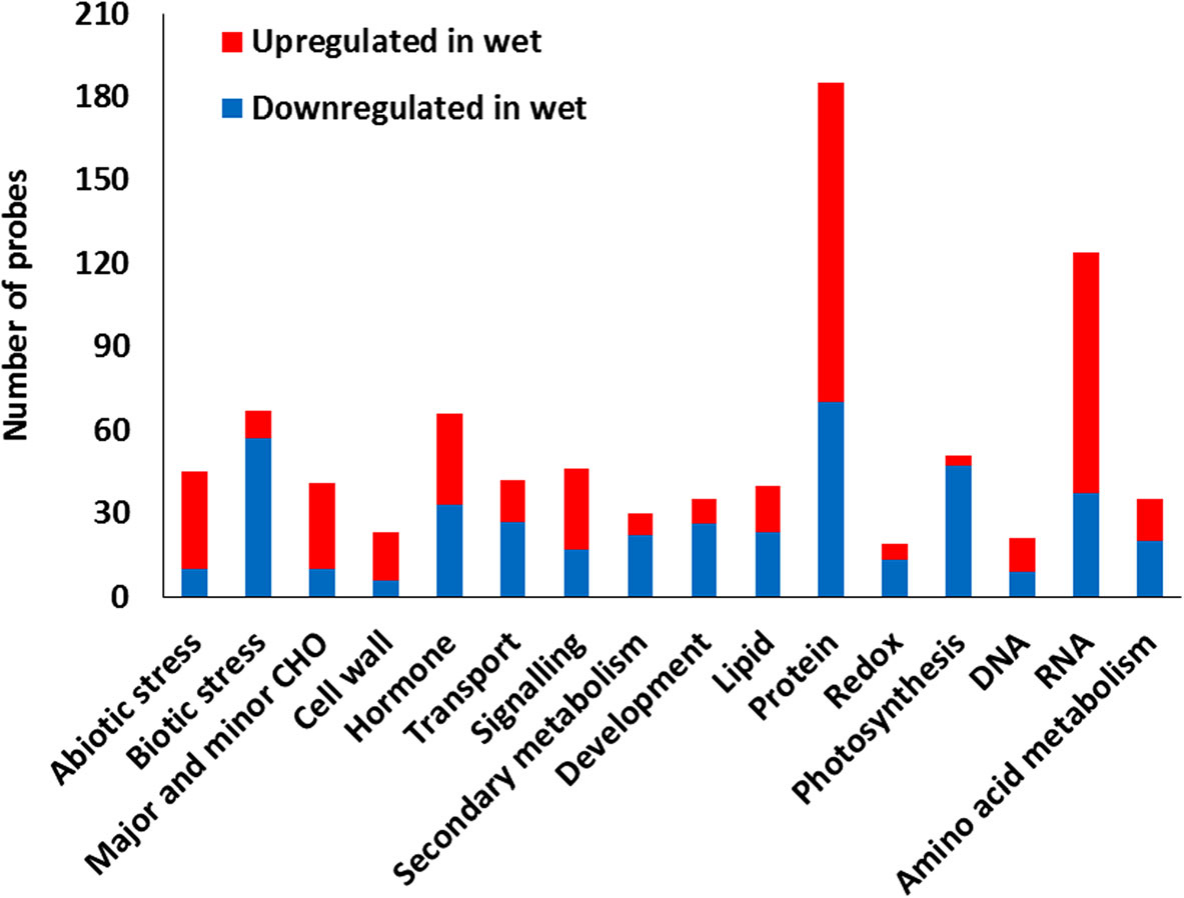
Top Gene Ontology (GO) Classes of up- and downregulated genes in microarray analysis. Expressed genes in potato tubers of cultivar ‘Bintje’ incubated one week in wet or dry conditions at 4 °C were compared. Log2 fold change of ≥ + 1 or ≤ − 1 and FDR value of ≤0.05 were used to select the differentially expressed genes. The y-axis indicates the number of probes in each function class, while the x-axis indicates the GO categories

The first transcriptome profiling was performed with a Potato Oligo Chip Initiative (POCI) microarray at 4 °C, a temperature used for long-term storage of ware potatoes. In total, 2165 differentially expressed genes (DEGs) were identified between the wet and dry treatments in potato flesh tissue after incubation during 1 week (w). Among the identified genes, 1057 genes were significantly upregulated, and 1106 were downregulated in the tubers incubated in wet conditions (Additional file 1). Gene ontology (GO) classes obtained from the POCI home page (http://pgrc.ipk-gatersleben.de/poci) showed that GO class had been assigned to 1080 DEGs. Among them, GO classes protein and RNA had the highest numbers of probes; furthermore, GO classes biotic stress and photosystem were downregulated, and abiotic stress was upregulated (Fig. 1). Closer look at the GO subclasses revealed that degradation and post-translational modification of proteins and regulation of transcription were represented by many probes, suggesting that wet conditions strongly affected stability and modification of proteins and transcriptional regulation of gene expression (Additional file 1).

**Fig. 2:**
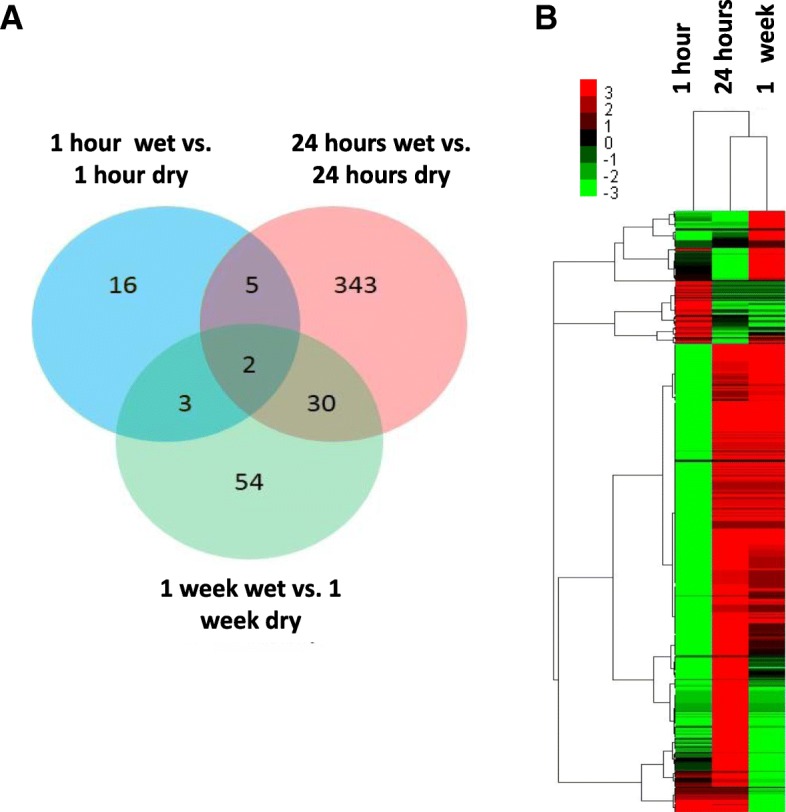
RNA-Seq profiling of potato tubers incubated in wet conditions at 15 °C. **a** Venn diagram of statistically significant differentially expressed genes (DEGs) in potato tubers incubated in wet conditions for 1 h, 24 h and 1 week shows that the responses at 24 h and 1 week are more similar to each other than those at 1 h. **b** Heat map of all the genes that were identified as statistically significant DEGs in at least one of the samples, based on clustering of 485 DEGs identified with NOIseq with probability > 0.8, *p*-value < 0.05 and log2 fold change ≥2 and ≤ − 2. Hierarchical clustering suggests that the 24 h and 1 week samples cluster together, while a difference was observed when compared to the 1 h sample. The scale bar at the top represents relative expression values. Red color indicates genes that were upregulated, and green color indicates genes that were downregulated. Black indicates genes whose expression is unchanged in wet tubers compared to dry tubers

To study the reaction of potato tubers to wet conditions at higher temperatures used for transport and pre-sprouting of seed tubers before planting, a second analysis was performed with RNA-Seq using tubers incubated at 15 °C. To observe also early processes, three time points were included: 1 h (h), 24 h and 1 w. In total, 45 million pure end pair reads from the Illumina Hiseq2000 sequencing platform were retrieved. GO classification was enriched on the identified DEGs, and a total of 460 unique GO functional annotation terms were determined in the 24 h comparison, while 51 and 14 unique GO functional terms were assigned for the 1 w and 1 h treatments, respectively (Additional file 2). Web Gene Ontology Annotation (WEGO) analysis suggested 7 significantly enriched (*p* < 0.05) GO functional annotation terms in the 24 h treatment, including peptidase inhibitor activity, peptidase regulator activity, enzyme inhibitor activity, oxidoreductase activity, endopeptidase inhibitor activity and endopeptidase regulator activity. This result is consistent with the downregulation of protease inhibitor genes in the microarray results of wet tubers. A detailed classification of KEGG pathways for the 483 DEGs of all three treatments was analyzed. In a 24 h comparison between wet and dry conditions, 174 DEGs were annotated to KEGG pathways, including 76 metabolic pathways. Of them, the significantly associated pathways (p < 0.05) were cysteine and methionine metabolism, glycolysis and gluconeogenesis, taurine and hypotaurine metabolism, and photosynthesis and antenna proteins, most of which were also identified in the results of the microarray that was performed at 4 °C. The DEGs were identified by NOISeq software and showed 26 DEGs in the 1 h sample, 380 in the 24 h sample and 89 in the 1 w sample (Additional file 2). The responses in 24 h and 1 w samples were more similar to each other than to the 1 h sample responses (Fig. 2a), which was also observed in hierarchical clustering (Fig. 2b). In general, high agreement between the microarray and RNA-Seq experiments was observed, suggesting that the response of potato tubers in wet conditions was similar at both temperatures (Additional file 3). When the two profiling experiments were compared, the most similar response was observed between the microarray results after 1 w of incubation at 4 °C and RNA-Seq results after 24 h at 15 °C, suggesting that the tuber response is faster at higher temperatures.

Comparison of low-oxygen-induced genes between organisms has led to the observation that almost half of the low-oxygen-induced genes in Arabidopsis code for unknown proteins, and 40% of them have upregulated orthologs in other plants, suggesting that the early responses of plants to low oxygen status and/or excessive water have not been well characterized [8]. Similar to Arabidopsis and other plants, in our results, there were many uncharacterized genes, especially in the 1 h sample, suggesting that the response of potato tubers to wet conditions and/or low oxygen in the early stage is also largely uncharacterized.

### Wet conditions caused a low-oxygen response and accelerated carbohydrate metabolism to compensate for energy insufficiency

In both microarray and RNA-Seq analyses, the expression of conserved genes representing the core low-oxygen response observed in many plants under hypoxic or anoxic conditions [8] was upregulated in the wet tubers, suggesting that the wet tuber tissues were suffering from reduced oxygen concentration compared to the dry tubers. The exposure of tuber surface to water has been shown before to lead to reduced oxygen uptake and anoxia that affect defense against pathogens [5]. Observation of low-oxygen response genes in the present study results suggests that the tubers reacted as expected; thus, the results can be used to understand how wet, low-oxygen conditions affect the other aspects of tuber physiology.

**Fig. 3:**
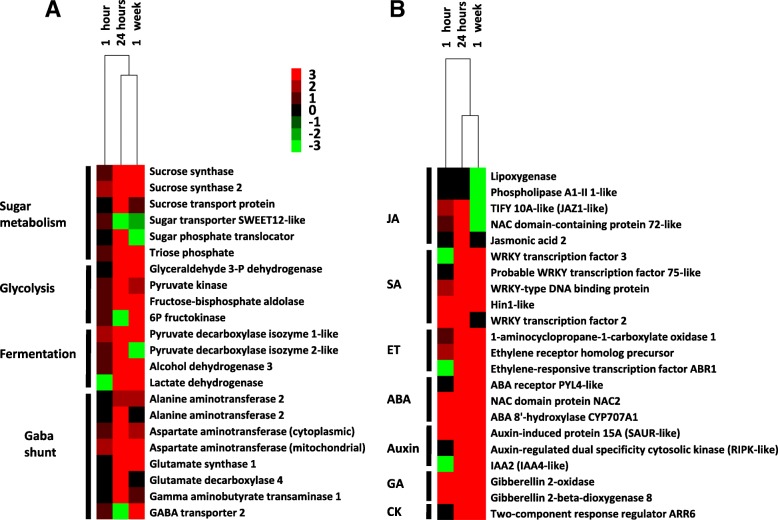
Heat maps with the primary RNA-Seq data of tubers incubated in wet conditions. **a** Heat map of sugar and amino acid genes show mostly upregulated gene expression. **b** Heat map of genes involved in phytohormone biosynthesis and signaling show downregulated gene expression linked to jasmonates and upregulated expression linked to other phytohormones. The rows represent statistically significant differentially expressed transcripts, and the columns depict three time-points. The rows are labeled with individual gene function, and the postulated function or hormonal pathway is indicated. The scale bar at the top represents relative expression values

The results of both transcriptome analyses suggested that carbohydrate and amino acid metabolism were altered in wet samples (Figs. 1, 3a), as observed before during waterlogging-induced hypoxia in green plants [8]. In the GO class major carbohydrate metabolism, genes involved in starch breakdown, such as plastid alpha-amylases and sucrose synthases, were upregulated in both analyses, and invertase transcripts were downregulated in wet conditions in the microarray, suggesting altered energy metabolism in wet conditions to protect tuber tissues against low oxygen levels [9]. Furthermore, genes in the GO class glycolysis were mostly induced during wet conditions in both analyses (Figs. 1, 3a). During wet incubation, the tuber starch is broken down by increased activity of amylases and sucrose synthases, and the glucose and other hexose phosphates are used in glycolysis to sustain energy to plant tissues during low-oxygen conditions [9]. The induction of alcohol dehydrogenase, pyruvate decarboxylase and lactate dehydrogenase transcripts was identified in both transcriptomes, suggesting fermentative energy production in wet conditions at both temperatures. Furthermore, the upregulation of genes that are involved in fermentation suggests that wet tubers produced energy by long-lasting ethanol fermentation rather than by rapid lactic fermentation that can impair cells due to acidification [10]. Genes included in the GO class minor carbohydrate biosynthesis suggested upregulated production of trehalose 6-phosphate (T6P), which is considered a signal regulating plant sugar metabolism, growth and development, possibly due to its interaction with sucrose nonfermenting (SNF) kinases [11]. In potato, T6P overproduction has been shown to cause the downregulation of cell proliferation and delayed growth and sprouting [12].

Genes involved in GO class amino acid metabolism were affected in both transcriptomes, most of which were upregulated, suggesting changes in amino acid biosynthesis in wet tubers (Figs. 1, 3a). Amino acid metabolism plays an essential regulatory role in the response of plants to various stresses. In recent years, several lines of evidence have shown the contribution of free amino acids to the energy-associated tricarboxylic acid cycle cycle upon exposure to various stresses that cause energy deprivation [13]. Differential expression of genes in gamma-aminobutyric acid (GABA) shunts, including the upregulated expression of glutamate decarboxylase and downregulation of GABA transaminase, suggest increased production of GABA, as observed before in hypoxic conditions in other plants [13]. GABA can act as a defense mechanism against oxygen deficiency by generating free alanine through the upregulation of alanine aminotransferase. Furthermore, glutamate decarboxylase induction regulates cytoplasmic pH because it consumes protons during GABA synthesis [14]. Alanine aminotransferase genes were upregulated in both analyses, suggesting increased alanine production, which was previously speculated to function as a storage form of excessive pyruvate. During the reoxygenation process, alanine can be transformed into pyruvate, which can enter the tricarboxylic acid cycle to produce ATP [15]. Furthermore, genes involved in protein degradation, ubiquitination and proteasome function were upregulated in wet tubers, suggesting that free amino acids may have been produced in the wet tubers also by the degradation of proteins [16].

In conclusion, wet tubers showed typical plant responses to low oxygen levels in hypoxic or anoxic conditions. This result could have been caused by two effects, either by reduced diffusion of air into the wet tubers, or an increased oxygen need of the wet tubers, which caused them to upregulate low-oxygen response genes earlier when compared to dry control tubers.

### Wet conditions enhanced oxidative stress and reduced the expression of chloroplast genes

Many DEGs in the microarray and RNA-Seq results were associated with the production of oxidative stress due to the production of ROS or were involved in antioxidant defense. Increasing evidence shows that ROS play crucial roles in low-oxygen responses, either as signaling molecules or as a cause of cell death [17]. Considering that potato tubers consist of nonphotosynthetic tissue, most ROS are generated in the mitochondrial electron transport system and plasma membrane by enzymes including amine oxidase, peroxidase, and NADPH oxidase as well as the key enzyme respiratory burst oxidase homolog, and representatives of all these genes were upregulated in wet tubers (Additional file 1, 2). The ROS burst in mitochondria may lead to a loss of outer membrane potential, which is an early marker for programmed cell death in *A. thaliana* [18]. Due to the detrimental effects of ROS, antioxidative defense systems, including a range of ROS scavengers such as superoxide dismutase, ascorbate peroxidase, catalase and glutathione S-transferases, are essential for the protection of cells against ROS [19]. Most of these genes were included in the data, suggesting that the ROS scavenging system was increased in the wet tuber tissue.

The results of both analyses indicated that heat shock protein (HSP) genes were induced in wet tubers, possibly protecting the proteins against ROS. Various HSPs were the main group of genes in the abiotic stress GO class in the microarray (Fig. 1). HSPs play key roles in the maturation of protein complexes, regulation of protein signal transduction and degradation of damaged or misfolded peptides. The HSPs are categorized according to their approximate molecular weight into five classes [20], all of which were found to be upregulated in microarray and/or RNA-Seq analyses. The high number of upregulated HSP genes suggests that wet, low-oxygen conditions may have detrimental effects on protein stability.

Almost all genes in the photosystem GO class, including photosystems I and II, were downregulated in the profiling results, and furthermore, many genes annotated as chloroplast precursors were among the downregulated genes, suggesting general downregulation of chloroplast functions in wet tuber tissue (Fig. 1, Additional files 1, 2). Repression of photosynthesis and chloroplast-associated genes has been observed in late stages of low-oxygen stress in many plants [21]. It has been suggested that one of the main strategies of plants to restrict ROS and to avoid cell death under abiotic stress is to reduce ROS formation by downregulating the synthesis of chlorophyll and other components of the photosynthetic machinery [22]. Potato tissues contain amyloplasts, but when the tubers are exposed to light, the plastids develop into chloroplasts, and the tubers turn green. Submergence of tubers in water has been experimentally used as a way to reduce tuber greening [23], supporting the observation that amyloplast to chloroplast change is prevented in wet tubers.

Contrary to downregulated expression of most plastid genes, plastidial acyl-[acyl-carrier-protein] (ACP) desaturases were upregulated in both analyses in wet tubers and in the RNA-Seq analysis this upregulation occurred already in the 1 h sample, which suggests a rapid response. ACP desaturases belong to the fatty acid desaturase family that introduce double bonds into fatty acids to produce unsaturated fatty acids. In Arabidopsis, similar genes were shown to be involved in drought and hypoxia stress signaling in crown galls [24]. Tuber ACP desaturase may be involved in the synthesis of oleic acid (18:1), which influences membrane fluidity and therefore plays a critical role in acclimation to environmental stresses and is also necessary for normal defense reactions [25].

### Cell wall integrity alterations, reduction in storage proteins and secondary metabolites in wet tubers may increase pathogen susceptibility

Both the microarray and RNA-Seq results showed that the GO class cell wall modification contained many differentially expressed genes (Additional files 1, 2). Genes involved in cellulose synthesis and degradation were mostly downregulated, whereas xyloglucan endo-transglycosylases, pectinesterases and expansins were upregulated in both analyses, suggesting changes in the cell wall structure because of wet conditions. Primary cell walls consist of cellulose that is cross-linked by hemicellulose molecules, such as xyloglucans and expansin, with pectins [26], and provide mechanical strength and form a structural barrier against pathogens. Our results suggest that the incubation of tubers in wet conditions upregulated enzymes that de-esterify pectin, possibly initiating cell wall loosening and allowing growth as well as making cell walls vulnerable to further pectinolysis, which in turn may enhance the susceptibility of the tubers to necrotrophic pathogens [27].

Many genes included in the GO class secondary metabolism were among the DEGs, suggesting changes in the secondary metabolites in wet tubers. Several genes involved in lignin biosynthesis were among the upregulated DEGs, whereas most flavonoid genes were downregulated. Furthermore, genes annotated as UDP-galactose:solanidine galactosyltransferase and rhamnose:beta-solanine/beta-chaconine rhamnosyltransferase, involved in the last two steps of α-solanine production [28], were downregulated in the microarray results, suggesting reduced production of glycoalkaloids in wet conditions. Submersion in water has been studied as a way to prevent glycoalkaloid accumulation in light-exposed tubers, but practical utilization of this method was prevented because the treatment also induced rotting [23].

A large group of downregulated genes, 70 in total, were annotated as protease/proteinase inhibitors (PIs) in the microarray result, where they comprised the major part of the biotic stress GO class (Fig. 1). Additionally, in the RNA-seq analysis, several protease inhibitors were identified; however, they were less clearly downregulated. The differentially expressed inhibitor-encoding genes were annotated as Kunitz-type PIs, serine PIs, cysteine PIs, aspartic PIs and metallocarboxypeptidase PIs. In addition to being storage proteins, PIs can play a potent defensive role against pathogens and insects [29]. Furthermore, 23 transcripts of patatin storage protein in the microarray included in the GO class development had reduced expression under wet conditions in the microarray, and patatin genes were also downregulated in the RNA-Seq results. PIs and patatins are present at high levels in stored and dormant potato tubers, whereas the downregulation of PI and patatin gene expression is linked to dormancy cessation and sprouting [30]. PIs are expressed as part of the wound response that is regulated by JA to protect wounded potato surfaces [31]. The downregulation of PIs and patatin genes may negatively affect the defense mechanism in wet tubers and increase susceptibility to necrotrophic pathogens during storage.

### Plant hormone-mediated regulation of the response to wet conditions

Both microarray and RNA-Seq profiling suggested altered phytohormone regulation in wet conditions (Figs. 1, 3b, Additional files 1, 2). Several lipoxygenases and allene oxide synthase were downregulated in 24 h or 1 w samples of wet-treated tubers, suggesting reduced biosynthesis of jasmonic acid (JA) or other jasmonates in wet tubers [32]. Furthermore, upregulated gene annotated as TIFY 10A in RNA-Seq was similar to the JA negative regulator JAZ1. In the microarray, a downregulated gene identified by the probe MICRO.15471.C1 was most similar in BLAST to a gene annotated as a bHLH transcriptional regulator, identical to potato MYC2, a positive regulator in JA signaling, which also suggests downregulation of JA signaling in wet tubers. In potato, anaerobic conditions inhibit the wound response that is regulated by JA [4], and in submerged Arabidopsis tissues, the JA concentration has been reported to be low [32]. Exogenous application of JA and other jasmonates improves plant resistance against necrotrophic pathogens and increases tolerance to abiotic stresses, such as drought, salt and chilling stress, suggesting that jasmonates have a role in both abiotic and biotic defense mechanisms [33].

Salicylic acid (SA) production has been suggested to be important for plant tolerance to oxidative stress [34], suggesting that SA production may be upregulated in wet tuber tissues. However, no clear changes in SA biosynthesis genes were observed in both analyses, whereas a Nim1 transcript similar to an NPR3-like gene possibly coding for an SA receptor, TGA2 and SA-induced NIMIN2-like and HIN1-like transcripts were upregulated in either one or both of the analyses, indicating active SA signaling in wet tubers.

The expression of ET biosynthesis and signaling genes coding for 1-aminocyclopropane carboxylate oxidases, ET receptors and ET-activated genes increased in wet tubers, suggesting high ET production by wet tubers (Figs. 1, 3b). ET has been linked to flooding and submergence response due to entrapment of the gaseous hormone in submerged tissues, and it is considered the main hormone regulating flooding and submergence response [3]. ET production activates genes involved in flooding tolerance, glycolysis and fermentation and induces adventitious root growth due to positive role of ET on auxin concentration and transport [35, 36]. ET may initially reduce dormancy in potato tubers, while prolonged exposure to ET is considered to prevent sprouting [37].

Both transcriptome results showed DEGs involved in abscisic acid (ABA) production in wet tubers. The expression of several aldehyde oxidases and a 9-cis-epoxy-carotenoid dioxygenase 1 transcript, possibly involved in the biosynthesis of ABA, showed downregulated expression, and the ABA repressor ABR1 was upregulated in the 24 h sample in the RNA-Seq analysis, both supporting the downregulation of ABA responses. The ABA receptor was upregulated in one-week sample. ABA biosynthesis has been observed to either increase or decrease in flooded plants, depending on the plant species and organ [35]. ABA is involved in defense against abiotic stresses and negatively affects JA signaling and resistance against necrotrophic pathogens [38]. In addition, ABA affects dormancy regulation in tubers [39], and as a consequence, ABA concentration change in wet tubers may affect dormancy, sprouting and pathogen defense.

Several probes in the microarray suggested induced auxin-related gene expression in the wet tubers. Genes coding for AUX/IAA proteins, markers of auxin and indole-3-acetic acid (IAA) response [40], early auxin responsive SAUR-like protein transcript and auxin-induced glutathione S-transferases were upregulated, whereas auxin response factors, auxin-repressed genes and genes coding for proteins involved in auxin transport were downregulated. In the RNA-Seq analysis 24 h sample, IAA2 transcript and an auxin-induced protein 15A-like, similar to SAUR, were upregulated, suggesting induced auxin production at 15 °C. During sprouting, auxin controls the growth of vascular tissue connection to the growing sprouts. In Arabidopsis, auxin controls adventitious root growth by downregulating JA signaling, which, in contrast, is able to inhibit auxin-induced lateral rooting, suggesting antagonism between auxin and JA signaling in root growth [41, 42]. Auxin production has been linked to reduced defense against plant pathogens, possibly due to a trade-off between growth and pathogen defense [43].

Only a few genes involved in gibberellin (GA) production were observed in the wet tubers. In RNA-Seq, GA catabolism gene GA 2-oxidase, GA inactivation gene GA 2-beta-dioxygenase and GA negative regulator ARR6-like gene were upregulated, suggesting GA degradation in wet tubers. Additionally, brassinosteroid biosynthesis appeared downregulated in wet tubers. Previous studies have indicated that GA is necessary for dormancy cessation in tubers and that BRs confer resistance to various abiotic stresses [44, 45].

In conclusion, wet conditions induced plant hormonal responses that most likely conferred resistance to low-oxygen conditions during treatment but also affected phytohormones associated with growth. Plant responses to abiotic and biotic stresses involve a complex crosstalk among phytohormones to increase survival under unfavorable conditions and to promote growth when beneficial. It seems that in the artificial environment of potato storage, the allocation of energy between defense and growth is not optimal for the survival of potato tubers.

### Senescence was induced in wet potato tubers

Several proteases in the GO class protein degradation, including serine and cysteine proteases and two chloroplast protein-degrading FtsH proteases, were upregulated, pointing to induced senescence in the wet tubers [46] (Additional file 1). Additionally, the massive downregulation of protease inhibitors, upregulation of ubiquitination, lipid degradation and ROS have been linked to senescence of green leaves. Furthermore, high trehalose-6P synthase (TPS) expression has been suggested as a marker for senescence [47]. At both temperatures, some genes, such as late embryogenesis abundant protein Lea5-like, also called senescence-associated gene 21 (SAG21), and wound-induced protein 1 gene (SAG20), were upregulated, supporting senescence in wet tubers [48].

Many phytohormones promote senescence in green leaves, most importantly ET, ABA and JA, whereas the role of auxin is not clear [46]. In potato tubers, senescence and degradation of tuber components occurs during sprouting, when the seed tuber resources are recycled for sprout growth. In contrast to green leaves, in potato tubers, both senescence and initiation of growth occur simultaneously, most likely causing different hormonal regulation compared to senescence of green leaves. In wet tubers, the upregulation of auxin may induce growth, while simultaneous ET production may cause senescence and prevent sprout formation, leading to nondormant but nonsprouting tubers with accelerated senescence and reduced defense to pathogens. Comparison of wet-induced genes in the present profiling to expression changes during dormancy termination and sprouting of tubers [49] and senescence in green leaves [50] shows similar transcriptomic changes, supporting the conclusion that dormancy, growth and senescence were affected in the wet tubers.

### Signaling and transcriptional regulator genes were involved in wet tubers to balance stress responses and growth

The characterization of the signaling genes and transcriptional regulators suggests that the potato tubers needed to adapt to abiotic stresses in wet conditions. Most of the genes in the GO class signaling, mainly represented by calmodulins and calmodulin-binding proteins, G-proteins, MAP kinases and receptor-like protein kinases, were upregulated in the microarray results. Similar signaling genes were upregulated in the RNA-seq results. With comparison to Arabidopsis genes, several of them were identified as regulators of ABA and auxin signaling, suggesting changed production of both hormones in wet conditions. Kinases in the GO class protein post-translational modification, such as Shaggy-like kinases, serine/threonine CBL-interacting protein (CIP) kinases and receptor-like kinases, involved in regulation of biotic and abiotic interactions and hormone and nutrient status of the plant, were also mostly upregulated. In other plants, several of the differentially expressed CIP and Shaggy kinases have been suggested to function as negative regulators of pathogen defense, as well as positive regulators of abiotic stress tolerance, root and xylem growth and floral development [51, 52].

In the GO subclass of transcriptional regulation, 95 genes divided into 34 families were identified, which was the largest group among GO classes in the microarray results. Most of the regulatory genes, especially those in the transcriptional regulators in NAC and MADS families, were upregulated. Comparison of these genes to Arabidopsis genes revealed similarity to several NAC regulators, among them ATAF1, a negative regulator of defense against necrotrophic pathogens, and VND1, a positive regulator of xylem development [53]. Furthermore, also several MADS box regulators, some annotated as AGL8 (POTM1) involved in suppression of meristem development and flowering or upregulation in roots in potato and other plants, were upregulated in wet tubers [54]. The WRKY transcription factors were upregulated especially in RNA-Seq data under wet conditions. When the identified potato WRKYs were compared to the function of the most similar ones in *Arabidopsis thaliana*, the WRKYs could be divided into two functional groups: WRKYs mainly involved in the negative regulation of pathogen defense and WRKYs mainly needed for abiotic stress defenses [55]. In conclusion, the regulatory changes in wet tubers supported upregulated abiotic defense mechanisms, reduced pathogen defense and changes in the growth-related processes in the wet tubers.

### Validation of profiling results with qRT-PCR

**Fig. 4:**
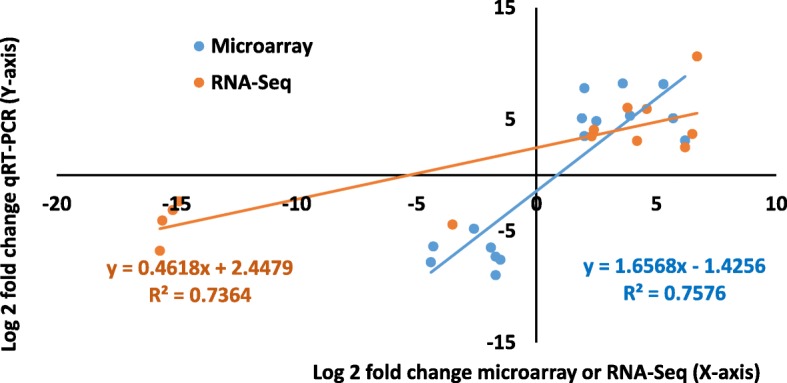
qRT-PCR validation of differentially expressed genes in wet potato tubers. Correlation coefficient analysis between microarray or RNA-Seq data (x-axis) and qRT-PCR (y-axis) data (log2 fold change) analyzed by the Pearson test (*P* < 0.05). The trend line equation and the corresponding strong square regression coefficient (R2) are shown. The classification of the genes and the primers used are presented in Additional file 4, and qRT-PCR data on the individual genes are presented in Additional file 5

To confirm the accuracy of the microarray and RNA-Seq data, qRT-PCR was performed for 28 DEGs from the microarray and RNA-seq results (Additional file 4). The genes for the primer design were selected to represent both highly or moderately up- or downregulated genes from different GO classes and processes, such as defense responses, sugar and amino acid metabolism, cell wall, secondary metabolism, hormones and signaling. The comparison of the expression levels of the selected genes between the profiling data and qRT-PCR (Additional file 5) were analyzed by Pearson coefficient correlation analysis to study the similarity in expression trends (up- or downregulation) between the profiling and qRT-PCR data (Fig. 4). The results showed that there was a strong correlation between the profiling methods and the qRT-PCR analysis data, suggesting that the results of both profiling methods could be verified with qRT-PCR.

### Proteinase inhibitor activity assay

**Fig. 5:**
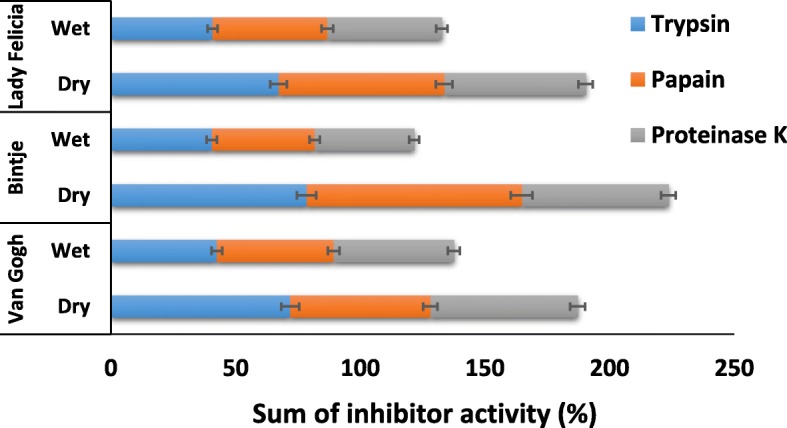
Reduction of proteinase inhibitor activity in potato tubers incubated in wet conditions. Three potato tuber cultivars were incubated in wet or dry conditions for one week. To measure the activity of the proteinase inhibitors, the potato extracts were incubated with three commercial proteinases, after which the proteinase activities were measured with the azocasein method. The percentages of protease inhibition were calculated by comparing the activities before and after incubation, with the negative control containing no potato extract, and calculated using the least significant difference test at *P* < 0.05 (*p* < 0001). The error bars represent the 95% confidence intervals for the estimated proportions representing four replicates of five tubers per protease assay

The downregulation of PI gene expression in wet conditions was a profound effect in the microarray results. To characterize the effect of the wet treatment on proteinase inhibitor activity, PI activities were measured in a separate experiment in three potato tuber cultivars stored in wet and dry conditions (Fig. 5). Protease inhibitor activities were calculated as percent of inhibition for comparative purposes using the least significant difference (LSD) test at *P* < 0.05. The assay showed that PI activities toward all tested proteases were reduced considerably (*p* < 0.0001) in wet tubers. Maximum inhibition percentage was shown by Bintje toward papain and trypsin, at 86.3 and 78.5%, respectively.

### Effect of ET inhibitor and MeJA on tubers incubated in wet conditions

**Fig. 6:**
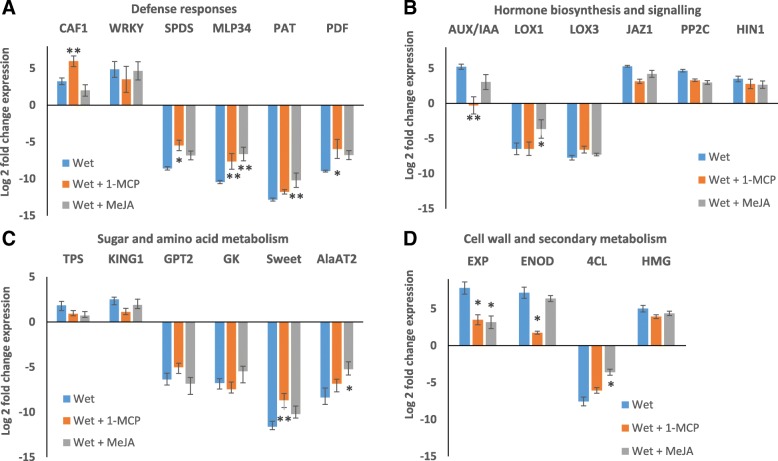
Effect of ethylene inhibitor and methyl jasmonate treatment on gene expression in wet potato tubers. Incubation of potato tubers in wet conditions in the presence of ethylene inhibitor 1-methylcyclopropane (1-MCP) or methyl jasmonate (MeJA) reduced the effect of wet conditions in qRT-PCR analysis. The chosen genes representing different functions were classified into four groups: **a** Defense responses: CCR4 associated factor 1-like (CAF1), WRKY-type DNA binding protein (WRKY), spermidine synthase 1-like (SPDS), major latex-like protein-like 34 (MLP34), patatin (PAT), defensin protein precursor (PDF); **b** Hormone biosynthesis and signaling: Nt-iaa4.5 deduced protein (AUX/IAA), lipoxygenase 1 (LOX1), lipoxygenase 3 (LOX3), protein TIFY 10A-like (JAZ1), protein phosphatase 2C 63 (PP2C), harpin inducing protein-like (HIN1); **c** Sugar and amino acid metabolism: Trehalose-6P synthase (TPS), SNF1-related protein kinase regulatory subunit gamma-1 (KING1), glucose-6-P translocator 2 (GPT2), glycerol kinase (GK), bidirectional sugar transporter (Sweet), alanine aminotransferase 2 (AlaAT2); **d** Cell wall and secondary metabolism: Expansin (EXP), early nodulin 93 (ENOD), 4-coumarate CoA ligase-like 2 (4CL), HMG-CoA reductase (HMG). The graph demonstrates means of log2 fold change from three independent experiments, error bars show standard error of mean and t-test results are shown (*p < 0.05, **p < 0.01)

As ET and JA levels are known to be affected in flooded or submerged plants, it was considered crucial to study the role of increased ET production and reduced JA production as mediators in the response of the tubers to wet conditions. In total, 24 genes were analyzed with qRT-PCR from wet tubers treated with ET inhibitor 1-methylcyclopropene (1-MCP) and methyl jasmonate (MeJA), and the values were compared to values obtained with tubers incubated in dry and wet conditions without treatment. Application of 1-MCP and MeJA reduced the differential expression of most of the analyzed genes when compared to the wet/dry ratio in the control tubers (Fig. 6). However, most genes were only slightly affected by the ET inhibitor or MeJA treatment, suggesting that these phytohormones are not the driving force for most changes observed in wet tubers. In addition, it was observed that 1-MCP did not have an effect on the tuber-specific LOX1 gene, suggesting that downregulation of JA-regulated genes in wet conditions was not caused by increased ET expression and its negative effect on JA production. The most drastic result in the qRT-PCR of 1-MCP treated samples was observed on the auxin response marker gene IAA/AUX. This gene was upregulated in wet tubers both in microarray and RNA-Seq experiments, but the upregulation was completely inhibited by 1-MCP treatment, suggesting that in wet conditions, ET production affected auxin production. In other plants, auxin and ET have been found to have both antagonistic and synergistic effects on root and seedling growth [56].

### Wet conditions affect hormonal balance and subsequent growth of potato tubers

**Fig. 7:**
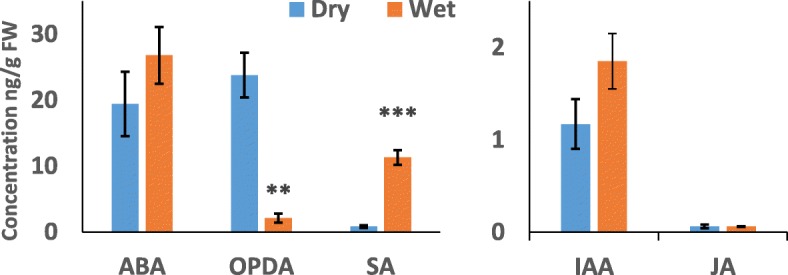
Phytohormone concentrations in wet and dry potato tubers. Concentrations of abscisic acid (ABA), 12-oxo-phytodienoic acid (OPDA), salicylic acid (SA), indole-3-acetic acid (IAA) and jasmonic acid (JA) were measured after incubation of the tubers for one week at 15 °C. The numbers are means of five samples each containing tissue from five tubers. Error bars show standard error and t-test values are shown (**p < 0.01, ***p < 0.001)

The profiling experiments indicated that a water film on the tuber surface initiates changes in the production of several phytohormones involved in growth and defense. To verify these results, the effect of wet conditions on IAA, ABA, SA, JA and the JA precursor 12-oxo-phytodienoic acid (OPDA) was measured in a separate experiment after one week of incubation of the tubers in wet conditions at 15 °C (Fig. 7). After incubation, wet tubers had significantly higher SA levels (*p* < 0.0005), possibly converted from glycosylated forms because no gene expression change could be linked to upregulated SA biosynthesis in wet tubers. Additionally, somewhat higher IAA and ABA levels were identified in the wet tubers when compared to the dry tubers. Both profiling experiments supported induced IAA production, and an induced IAA production was also observed in the hormone measurements in wet samples. In the case of ABA, the results of the profiling and hormone measurement were contradictory, making it difficult to know how ABA concentration was affected in the wet tubers. It is possible that the ABA level was reduced at 4 °C as suggested by the microarray results, while at 15 °C, the ABA concentration increased later in the 1 w samples, as suggested by the ABA measurement. Only low JA amounts were identified in the samples, but the amount of JA precursor OPDA was reduced (*p* < 0.005) in the tubers incubated in wet conditions, supporting the downregulated jasmonate production observed in both profiling results. Antagonism between SA or auxin and the JA precursor OPDA may cause the observed reduction in JA-related gene expression and pathogen defense in wet tubers [4].

**Fig. 8:**
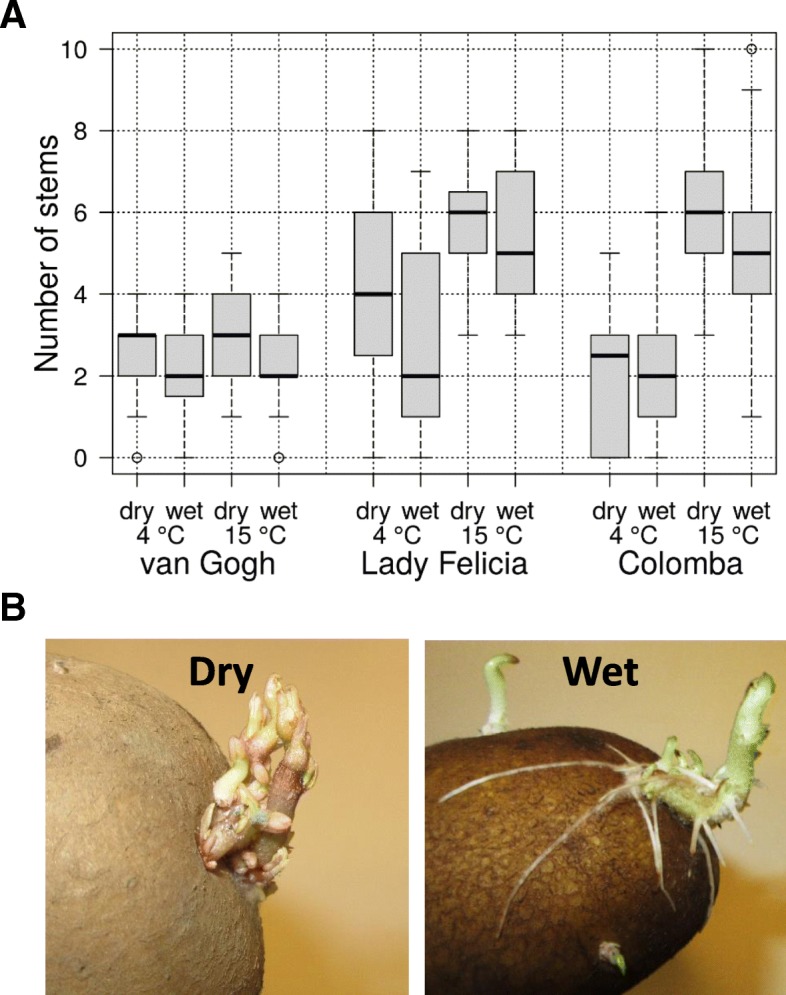
Sprouting and growth of potato tubers after incubation in wet and dry conditions. **a** Statistical analysis of stem numbers of non-sprouted potato tubers of three cultivars after incubation for one week in wet or dry conditions at 4 °C or 15 °C. Both temperature (*p* < 0.0001) and incubation in wet conditions (*p* = 0.003) had a significant effect on the number of stems during growth of the plants in the greenhouse. **b** Wet incubation of sprouted tubers at 22 °C led to root growth, while the control tubers incubated in dry conditions did not show any root growth

To characterize the effect of changes in gene expression and phytohormone concentrations on the growth of the tubers, potato tubers of three cultivars were incubated in wet or dry conditions for a week at 4 °C or 15 °C and then planted in a greenhouse to grow in + 22 °C. There were no phenotypic differences in the growing stems; however, the tubers that had been incubated in wet conditions consistently developed fewer stems per tuber (Fig. 8a). Both conditions during incubation (wet or dry) and temperature (4 °C or 15 °C) had statistically significant effects, incubation condition with *p* = 0.003 and temperature with *p* < 0.0001 over the whole experiment. The effect of the wet conditions on the number of stems may be caused by changes in dormancy that is regulated by phytohormones [44]. During dormancy release, apical dominance is reduced, leading to an increase in the number of developing stems. Increased ABA concentration may lead to delayed loss of dormancy in wet tubers, which would lead to a reduced number of developing sprouts. Delayed dormancy cessation may also be caused by an increase in T6P concentration that delays sprouting and growth in potato, and furthermore, a high sucrose level has been indicated in dormancy release [39], and it is possible that the wet, fermenting tubers have a high T6P concentration but low sugar content, leading to slower dormancy release and fewer developing sprouts.

To find phenotypic changes linked to auxin production, tubers with 1–2 cm sprouts were exposed to wet and dry conditions at 22 °C to mimic the greenhouse conditions (Fig. 8b). The wet conditions induced growth of adventitious roots in the sprouting tubers during one week of incubation in all incubated tubers, whereas no roots developed under the dry conditions. These results suggest that incubation in wet conditions may cause a change in the hormonal balance that promotes root growth. In tomato and rice, ET and auxin interact to promote adventitious root formation in submerged or flooded plants [57], suggesting that water-induced adventitious root growth is initiated similarly in wet tubers and flooded or submerged plants. Furthermore, for seeds of many plant species, the presence of water causing imbibition of the seeds is needed for germination and primary root growth [58]. It is possible that for potato tubers, the exposure of the tuber surface to water is also a signal for root growth.

## Conclusions

**Fig. 9:**
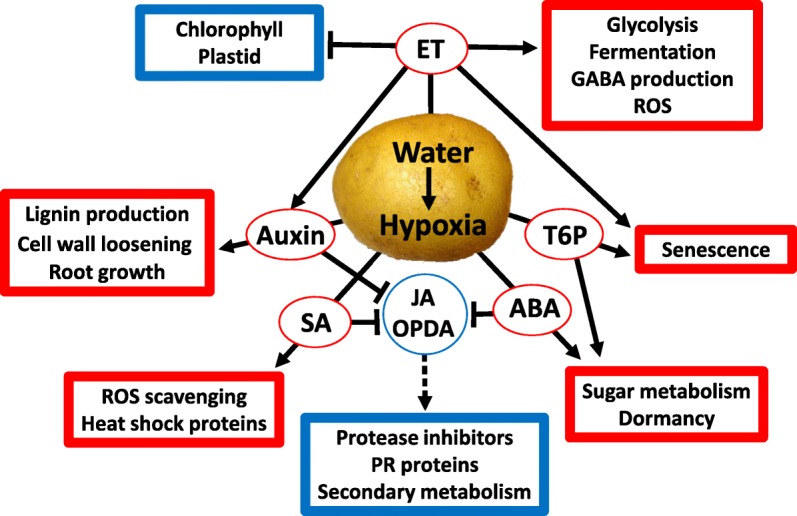
Model of the main identified signaling pathways involved in the response of potato tubers to wet conditions. The main groups of differentially expressed genes in potato tubers in wet conditions (upregulated, red; downregulated, green) and the postulated hormones affecting their expression are indicated. T6P, trehalose 6-phosphate; ET, ethylene; SA, salicylic acid; JA, jasmonic acid; OPDA, 12-oxo-phytodienoic acid; ABA, abscisic acid; and IAA, auxin are indicated. Solid arrows indicate upregulated effect, blocked arrows suggest negative effect and dashed arrow indicate reduced jasmonate signalling in wet tubers

Transcriptome analyses were carried out in wet and dry potato tubers under low-oxygen conditions to understand the effect of water on stored tuber gene expression and physiology. The wet conditions reduced oxygen availability and led to low-oxygen stress that is typically seen in anaerobic or hypoxic conditions in flooded or submerged plants and in anoxic potato tubers. The reduced oxygen availability caused an energy crisis, which the tubers compensated for by the production of energy through glycolysis and fermentation, downregulation of secondary metabolism and degradation of starch and storage proteins to recycle sugars and amino acids (Fig. 9). Wet, low-oxygen conditions are an abiotic stress leading to ET and ROS production, which the tubers tackled with increased production of HSPs and ROS scavengers and reduction of chlorophyll-related gene expression. Changes in phytohormones, especially increased SA and auxin, which are antagonistic to JA and may lead to downregulation of JA-dependent defenses, may render tubers susceptible to necrotrophic pathogens. Increased production of auxin may lead to cell wall loosening and increased lignin production, which indicate changes in growth-related processes in wet conditions, and it is also evidenced as induced adventitious root growth observed in sprouting tubers incubated in wet conditions. Increased TPS and possibly also ABA concentration under wet conditions may lead to slower dormancy termination that reduces stem number later during the growth of the sprouting tubers in the soil. It appears that wet tubers invest in root growth and defense against low oxygen levels and ROS, which can lead to reduced defense against necrotrophic pathogens. It is also likely that water on the tuber surface is an indication of suitable growth conditions and thus functions as a signal for energy allocation for root growth through induced auxin production. It appears that in wet, low-oxygen conditions, when the tuber energy level is low, the increased energy demand for growth and ROS defense may lead to reduced allocation of energy for pathogen defense. In addition to consequences for the storability of the potato tubers, wet storage conditions before planting may affect growth vigor of potato seed tubers during the following growing season.

## Method

### Plant materials and treatments

Potato tubers of cv. ‘Bintje’ were obtained from The Finnish Seed Potato Centre Ltd. for the profiling experiments, and for the other experiments all the potato tubers were purchased from a wholesale company (H&H Tuominen Ltd.). All the tubers were stored in cold room at 4 °C before the experiments. The profiling experiments were performed in January–March, when tubers have lost endodormancy and can be induced to sprout in suitable environmental conditions. The greenhouse experiments were performed on Viikki campus at the University of Helsinki in Finland.

Potato cv. ‘Bintje’ was used in the microarray profiling experiment to compare tubers incubated in wet and dry conditions. The potato tubers were washed thoroughly in tap water, surface sterilized by immersion in 3% sodium hypochlorite for 5 min and then dried for 45 min at room temperature in the dark prior to the treatments, after which the tubers were wrapped in wet paper towels and placed in plastic bags, each containing 20 tubers. To preserve the humidity in the bag containing the wet tubers, it was necessary to close the bags, which prevented air supply creating low-oxygen conditions. Control tubers were wrapped in dry tissue paper and incubated in closed plastic bags. The tubers were incubated in the dark at 4 °C for 1 w in the microarray experiment before sample preparation. The RNA-Seq experiment was performed at 15 °C, and samples were collected at 3 time-points: 1 h, 24 h or 1 w. After incubation, approximately 1 kg of potatoes, corresponding to 5–7 medium size tubers, was pooled for each replicate. To minimize contamination, periderm (skin) was separated with a sterilized scalpel, and three cubes (approximately 5 mm) were cut from the cortex, immediately frozen in liquid nitrogen and then kept at − 80 °C prior to RNA isolation. The sampling method outlined above was used for microarray, RNA-Seq, qRT-PCR and phytohormone analyses, each in a separate experiment. Three biological repetitions and three technical replicas, each a mixture of 5–7 tubers, were performed for each treatment.

To check the expression of selected genes under wet treatment after exposing potato tubers to MeJA or 1-MCP, an experiment was carried out with potato tubers of cv. ‘Bintje’ in three individual replicates. Approximately 5–7 surface sterilized tubers were placed in 4 l (l) gas-tight polycarbonate containers. 1-MCP was applied at a calculated final concentration of 800 nl/l by dissolving 4.5 mg of a powder containing 0.14% 1-MCP as active ingredient (SmartFresh™) in 5 ml of warm water (at 35 °C), and the container was sealed immediately. MeJA (Sigma) at 1 mM was sprayed on the tuber surface. The tubers were subjected to the following treatments: dry (control), wet, wet plus 1-MCP and wet plus MeJA, and then they were stored in the dark at 15 °C for 1 week. A sample was taken from two sides of the tuber and immediately frozen with liquid nitrogen and stored at − 80 °C for subsequent analysis. The experiment was performed twice.

To characterize the PI activity after wet incubation, the tubers of three cultivars, ‘Bintje’, ‘Van Gogh’ and ‘Lady Felicia’, were incubated in wet and dry conditions for one week in 4 l plastic containers at 15 °C, and then their PI activity was assayed. After incubation in wet and dry conditions, the tubers were sliced into large pieces (max 6 × 4 cm) and subsequently mixed in the presence of sodium bisulfide at a dosage of 0.5 g/kg of tuber to prevent oxidation of phenolic compounds. The sample pieces were ground in a commercial blender, and starch was allowed to sediment for 30 min at 4 °C. The solution was centrifuged (10,000×g, 10 min, 4 °C), and the clear supernatant was assayed for PI activity.

To characterize the effect of wet and dry incubation of tubers on their subsequent growth, tubers of cv. ‘Van Gogh’, cv. ‘Lady Felicia’ and cv. ‘Colomba’ were sprayed wet with tap water or left dry, and 10 medium-sized tubers were closed in 4 l plastic containers at 4 °C or 15 °C for one week. The experiment was performed twice with 10 tubers/temperature for most cultivars and temperatures, but cv. ‘Colomba’ was used three times at 15 °C and once at 4 °C. In total, 240 tubers were treated, half of them in wet and half in dry conditions. The treated tubers were planted immediately after opening the boxes in 3 l pots in a greenhouse at 22 °C, 55% relative humidity and 18 h light and 6 h dark conditions. When the stems were at a minimum of 50 cm high, the number of stems growing from each tuber was counted. Stem count data from 240 tubers were analyzed with the R glmm package [59]. Stem count was used as a response variable in a Poisson regression model using incubation conditions (wet, dry) and temperatures (4 °C, 15 °C) as fixed effects and storage boxes (12 levels) as well as potato variety (3 levels) as random effects. Model parameters were estimated using 100,000 MCMC iterations.

To compare the effect of wet and dry incubation conditions on sprouting tubers, wet and dry tubers of cv. ‘Van Gogh’ and cv. ‘Lady Felicia’ with 1–2 cm sprouts were incubated in closed boxes at 22 °C for 1 w, the presence or absence of roots was observed and recorded, and the tubers were photographed immediately after opening the boxes. Each box contained 10 tubers, and the experiment was performed twice with both cultivars.

### RNA extraction

Total RNA was extracted directly from dissected frozen potato tuber tissue using Trizol reagent (TRIzol reagent, Ambion, Life Technology). To remove any trace of DNA and other possible contaminations, a TURBO DNA-free kit (Ambion) was used for DNase treatment, followed by an RNeasy MinElute clean up kit (Qiagen), all according to the manufacturer’s instructions. RNA concentration was quantified by A260 using a NanoDrop spectrophotometer (Thermo Scientific NanoDrop 2000). RNA integrity was assessed using 1% TAE-agarose gel electrophoresis evaluating the presence of intact ribosomal RNA bands. In the used experimental conditions, the tuber tissue did not show rotting and no degradation of plant mRNA was observed. Each sample was pooled by mixing equal quantities of total RNA from five technical replicates representing 5–7 tubers each per treatment and time-point. This isolation method was used to extract pure total RNA for microarray, RNA-Seq, qRT-PCR, MeJA and 1-MCP experiments.

### Microarray experiment and data analysis

The POCI array obtained from Agilent contains 44 k 60-mer oligos, which have been designed based on 46,345 unigenes and constructed from 246,182 expressed sequence tags (http://pgrc.ipk-gatersleben.de/poci). Total RNA was reverse transcribed into double strand cDNA using reverse transcriptase (Promega) with random hexamers and Cy3, Cy5 and Alexa488 (GE Healthcare). RNA was hydrolyzed by adding 0.1 M NaOH, and the samples were neutralized with 0.1 M HCl. After incubation at 37 °C for 3 h, the reactions of two samples (wet and dry) were combined and purified by using the Qiagen PCR purification kit, lyophilized, dissolved in 14 ml of hybridization buffer, denatured and loaded onto coverslips (Agilent). The slides were placed in a waterproof hybridization chamber, and hybridization was carried out overnight in a 65 °C water bath. After hybridization, the slides were washed three times (each 2 min), following the manufacturer’s instructions (Agilent), and were scanned on the Agilent Microarray scanner.

The dried microarrays were scanned with a 5 μm pixel resolution (Axon 4200 AL, Axon Instruments). To minimize the number of saturated pixel values, photomultiplier tube (PMT) gains were individually set for each array and fluorescence dye. Spot intensities were quantified by using Axon GenePix 6.0 software. To ensure optimal spot recognition, the grids were manually adjusted, and spots with dust or locally high background were discarded. Limma (Linear Models for Microarray data), R/Bioconductor software package, was used for normalization and statistical analysis. After subtracting the background median value from the spot median value, quantile normalization was applied to each sample, and a moderated t-test was performed to analyze the expression of genes under two conditions, dry and wet. To determine significant differences in gene expression, a log2 fold change of ≥ + 1 or  ≤ − 1 and FDR value of ≤0.05 were used.

### RNA-Seq library construction, sequencing and data analysis

A high-throughput (HT) Tru-Seq RNA Sample Preparation protocol (Illumina) was used according to the manufacturer’s instructions for 18 samples, three repeats in dry and wet conditions, each at three time points: 1 h, 24 h and 1 w. Individual samples were barcoded using different indexing adapters prior to quality check, pooling and sequencing. The quality and quantity of DNA were estimated by Agilent Technologies 2100 Bioanalyser using D1000 screen tape (Agilent). The libraries were sequenced to generate 100-bp paired-end reads on a Hi-Seq 2000 sequencer (Illumina) at the Institute of Chemistry and Biosciences, Aalborg, Denmark. Following sequencing and base calling using standard Illumina workflow, raw RNA-Seq data were obtained in FASTQ format. Reads with adapters, low quality sequences and poly-N reads were filtered by FastQC (http://www.bioinformatics.babraham.ac.uk/projects/fastqc). The Potato Genome Sequencing Consortium (PGSC) sequence of the doubled monoploid *S. tuberosum* Group Phureja DM1–3516 R44 with Genome Annotation v3.4 (PGSC_DM_v4.03) was used as reference data (http://potato.plantbiology.msu.edu/pgsc_download.shtml). The index of the reference genome was built by Bowtie v2.1.0, and the reads were mapped to the reference using TopHat v2.0.9 software. The mapping parameters were “-p 8 --b2-very-sensitive --solexa-quals --segment-length 30 --segment-mismatches 3 --mate-std-dev 20 --library-type fr-unstranded” (http://ccb.jhu.edu/software/tophat/index.shtml). The expression level of each transcript was determined based on the fragments per transcript kilobase per million fragments mapped (FPKM) value. DEGs between the control and treated samples were identified using NOIseq using read counts with probability> 0.8, *p*-value< 0.05 and log2 fold change ≥2 and ≤ − 2 [60, 61].

To visualize the gene expression, hierarchical cluster analysis was carried out by Cluster 3.0 and visualized using Java TreeView software. The parameters used in Cluster 3.0 were “-g 7 -e 7 -m a” (http://bonsai.hgc.jp/~mdehoon/software/cluster/software.htm). Functional annotation of genes and analysis of annotation data were performed by blastx analysis against the NCBI protein database, retrieving all hits below an E-value of 1e-5, and employing Blast2GO software (https://www.blast2go.com/). WEGO tools and KEGG pathway enrichment (http://www.genome.jp/kegg/pathway.html) were used for classification of GO functions and pathways.

### qRT-PCR analysis

To verify the microarray and RNA-Seq results, qRT-PCR was used to determine the target gene expression level in wet and dry samples. In the microarray data, the probe name (unigene ID) was used to retrieve unigene sequences from the POCI array site (http://apex.ipk-gatersleben.de/apex/f?p=194:1); in the RNA-seq data, the DMT number (such as PGSC0003DMT400039410) was used to retrieve the transcript sequence from the transcript reference file (PGSC_DM_v3.4_transcript_representative.fasta.zip) from the SOL genomic network. PrimerQuestSM (http://www.eu.idtdna.com/Primerquest/Home/Index) from Integrated DNA technologies was used to design the gene-specific primers for the selected genes. Primer pairs with corresponding sequences used for selected genes of microarray and RNA-Seq are presented in Additional file 4.

To make cDNA for qRT-PCR, clean total RNA was reverse transcribed using a SuperScript® VILO™ cDNA Synthesis Kit (ThermoFisher) and Enhanced Avian Reverse Transcriptase (Sigma) following the manufacturer’s instructions. The qRT-PCR experiments were performed three times with independent cDNAs using LightCycler® 480 SYBR Green I Master (Roche) according to the supplier’s instructions. For automation, all PCRs were pipetted using an epMotion® 5075 pipetting robot (Eppendorf), and qRT-PCR experiments were performed on a LightCycler® 480 Real-Time PCR System (Roche).

To verify the specificity of the primers, the presence of a single peak in the melting curve was observed. To verify amplification of a single product with the expected length, the amplicon of each target gene was visualized on a 1% agarose gel. The *S. tuberosum* eukaryotic translation initiation factor 5A, actin and potato starch phosphorylase genes [62] were tested as reference genes, and due to small variation between them, eukaryotic initiation factor 5A was chosen as a housekeeping gene. The relative fold change of target gene expression was normalized to initiation factor 5A and then calculated by 2-∆∆CT based on the Livak method [63]. The qRT-PCR experiments were performed three times with three technical replicates of each cDNA.

### Proteinase inhibitor activity assay

The inhibition potential of potato extract on the proteolytic activity of three commercially available proteinases, trypsin (EC.3.4.21.4, Product No. T8003, Sigma-Aldrich), papain (EC.3.4.22.2, Product No. P3375, Sigma-Aldrich) and proteinase K (EC.3.4.21.64, Product No. P2308, Sigma-Aldrich), was calculated by comparing with samples containing protease but without potato extract. A proteinase inhibitor cocktail (EC.3.4.21.64, Product No. P9599, Sigma-Aldrich) and water were used as positive and negative control samples, respectively. The proteolytic activity was quantified by azocasein assay according to the protocol of Brock et al. [64] with slight modifications. This assay is based on the hydrolysis of azocasein (Sigma-Aldrich) by proteases, resulting in the release of azo-molecules with a unique absorption at 436 nm. The assay was modified as follows. In a 2-ml Eppendorf tube, a 30 μl aliquot of commercial protease (10 mg/ml prepared in 1 mM HCl) was preincubated with 1.5 ml of potato extract at 20 °C for 1 h, and then 450 μl of azocasein solution (2.5%, prepared in 0.1 M Tris buffer pH 8) was added and incubated at 30 °C for 30 min. The reaction was terminated by adding 375 μl of 14% perchloric acid (Sigma-Aldrich) to precipitate the proteins. The assay mixture was centrifuged at 8000×g for 10 min, and 375 μl of the supernatant was added and mixed with 75 μl of 10 mM NaOH. Then, 350 μl of solution was placed in a spectrophotometer cuvette (10 mm), and the absorbance was measured at 436 nm using a NanoDrop spectrophotometer. There were four replicates of five tubers per protease treatment (dry and wet) for each potato cultivar, and the experiment was independently repeated. The percentage of inhibition was measured using the following formula: inhibition percentage = (negative sample-inhibited sample)/negative sample) × 100. Data were analyzed for variance by ANOVA using the general linear model of the SAS statistical package (V 19.0), and the means were separated using LSD test at *P* < 0.05.

### Extraction and quantification of phytohormones

Phytohormones ABA, IAA, SA, JA and JA precursor OPDA were analyzed from wet and dry tubers incubated for a week at 15 °C. Approximately 400 mg (FW, fresh weight) of snap frozen, ground tuber samples was extracted with 1 ml of an ice cold (− 20 °C) methanol: isopropanol: acetic acid (20:79:1) mixture twice [65]. Five microliters of internal standard mix (ISTD; SA-d4, dh-JA, ABA-d6, IAA-d5; 100 ng/ml) was added. The extracts were evaporated to dryness and reconstituted in 50 μl acetonitrile (ACN) and then run immediately in randomized order with Ultra performance liquid chromatography - tandem mass spectrometer (UPLC-MS). Five replicate samples, each containing ground tissue from five wet or dry tubers, were analyzed in these experiments. The results of SA, JA, OPDA, ABA and IAA were normalized to the corresponding deuterated ISTDs (SA-d4, dh-JA, DnOPDa-d5, ABA-d6 and IAA-d5, respectively) and FW and were quantified using calibration curves for each phytohormone with Analyst MultiQuant™ software (ABSciex Pte. Ltd.).

The UPLC-MS system consisted of ExionLC UPLC connected to Sciex QTRAP-6500+ via ESI (AB Sciex Pte. Ltd.) The mobile phase consisted of 0.1% formic acid in MQ water (A) (Merck Millipore) and ACN (B) (Honeywell, Riedel-de Haën, CHROMASOLV™, LC-MS grade). The chromatographic separation was performed in a Waters Acquity UPLC BEH C18 (Ø 1.7 μm, 2.1 mm × 50 mm) column (Waters) with a flow rate of 0.6 ml/min and a linear gradient of 5 to 75% B in 7 min. The injection volume was 5 μl. The mass spectrometer was operated in multiple reaction mode (MRM) with polarity switching (ESI+/−) using optimized precursor-to-product ion transitions due to its high selectivity and increasing sensitivity for low abundance phytohormones. The MRMs used for each phytohormone and their corresponding ISTDs were 136.84 > 92.9 (ESI-) for SA, 140.88 > 97.0 (ESI-) for SA-d4, 208.95 > 59.0 (ESI-) for JA, 210.97 > 58.9 (ESI-) for dh-JA, 291.04 > 165.1 (ESI-) for OPDA, 268.03 > 170.1 (ESI-) for DnOPDA-d5, 262.96 > 153.0 (ESI-) for ABA, 269.01 > 159.0 (ESI-) for ABA-d6, 176.01 > 130.0 (ESI+) for IAA and 181.02 > 134.0 (ESI+) for IAA-d5.

## Additional files

Additional file 1: Microarray data. Raw data and DEGs of the microarray data with log2 fold change ≥ − 1 and ≤ + 1 and *p*-value ≤0.05, and categorized genes. (XLSX 6000 kb)

Additional file 2: RNA-Seq data. Raw data and DEGs of RNA-Seq data for one hour, 24 h and one week. DEGs identified with NOIseq with probability > 0.8, *p*-value < 0.05 and log 2 fold change ≥2 and ≤ − 2. Also the list of DEGs and categorized genes. (XLSX 1923 kb)

Additional file 3: Similarity between the microarray analysis at 4 °C and RNA-Seq analysis at 15 °C. (XLSX 24 kb)

Additional file 4: Primers used in this study. Primers for 15 DEGs identified from microarray and 13 DEGs from RNA-Seq profiling, including the target genes, abbreviations, GO class, suggested function and primer sequences. (DOCX 18 kb)

Additional file 5: Comparison of RNA-Seq and microarray analysis with qRT-PCR validation assays. Quantitative measurement of gene expression was determined with qRT-PCR for 15 DEGs in microarray (A) and 13 DEGs in RNA-Seq results (B). Data were obtained from three independent cDNA sets from three independent experiments, normalized to eukaryotic elongation factor 5A3 and expressed as the means of log2 (ΔΔCt) ± SEM (standard error of the mean). The statistical analysis for the data was performed with coefficient correlation analysis between microarray or RNA-Seq data and qRT-PCR data (log2 fold change) analyzed by the Pearson test (*P* < 0.05), which resulted in strong correlation between the analysis methods as indicated in Fig. 4. (PDF 180 kb)

## Data Availability

All data generated or analysed during this study are included in this published article and its additional files.

## Abbreviations

1-MCP: 1-Methylcyclopropane
4CL: 4-Coumarate CoA ligase
ABA: Abscisic acid
ACN: Acetonitrile
ACP: Acyl-carrier-protein
AlaAT: Alanine aminotransferase
AUX/IAA: Auxin/IAA
CAF: CCR4 associated factor
CIPK: CBL-interacting protein kinase
Cv.: Cultivar
DEG: Differentially expressed gene
EF: Eukaryotic elongation factor
ENOD: Early nodulin
ET: Ethylene
EXP: Expansin
FPKM: Fragments per transcript kilobase per million fragments mapped
FW: Fresh weight
GA: Gibberellin
GABA: Gamma-aminobutyric acid
GK: Glycerol kinase
GO: Gene ontology
GPT: Glucose-6-P translocator
HIN: Harpin inducing protein
HMG: HMG-CoA reductase
HSP: Heat shock protein
IAA: Indole-3-acetic acid
ISTD: Internal standard
JA: Jasmonic acid
JAZ: Jasmonate ZIM-domain protein
KING: SNF1-related protein kinase regulatory subunit gamma
Limma: Linear Models for Microarray data
LOX: Lipoxygenase
MeJA: Methyl jasmonate
MLP: Major latex protein
MRM: multiple reaction mode
OPDA: 12-Oxo-phytodienoic acid
PAT: Patatin
PDF: Defensin
PGSC: Potato Genome Sequencing Consortium.
PI: Protease inhibitor
POCI: Potato Oligo Chip Initiative
PP2C: Protein phosphatase 2C
qRT-PCR: Real-time reverse transcription PCR
ROS: Reactive oxygen species; SA: Salicylic acid
SAG: Senescence-associated gene
SNF: Sucrose non fermenting
SPDS: Spermidine synthase
T6P: Trehalose 6-phosphate
TPS: Trehalose-6P synthase
UPLC-MS: Ultra performance liquid chromatography - tandem mass spectrometer
WEGO: Web Gene Ontology Annotation Plot
