# Supplementary material for: Effect of wet storage conditions on potato tuber transcriptome, phytohormones and growth
Source: BMC Plant Biol. 2019 Jun 17;19:262. doi: 10.1186/s12870-019-1875-y (PMC6580497; doi:10.1186/s12870-019-1875-y)
Supplement: Supplementary file 4 — Primers used in this study. Primers for 15 DEGs identified from microarray and 13 DEGs from RNA-Seq profiling, including the target genes, abbreviations, GO class, suggested function and primer sequences. (DOCX 18 kb) [file 12870_2019_1875_MOESM4_ESM.docx]

|  | **Annotation** | **Gene** | **GO class** | **GO subclass/Suggested function** | **Primer sequences** |
| --- | --- | --- | --- | --- | --- |
| 1 | Lipoxygenase 1 | LOX1 | Hormone | Fatty acid biosynthesis, jasmonate biosynthesis | Forward: CCCTGAATTACATGCCCTGTTTGATG  Reverse: GGAAGTTTGATCCCTCCTTCATAT |
| 2 | Expansin | EXP | Cell wall | Cell wall modification, growth | Forward: GTGGAGCATGTTGGATTGGTGAATATG  Reverse: ACCATCCCGTTCCATTTCCTGTAG |
| 3 | 4-Coumarate CoA ligase-like 2 | 4CL | Secondary metabolism | Phenylpropanoids, lignin | Forward: GATTTCAAGTGGCTCCTGCTGAAC  Reverse: GCTTGCTCGTCTTTCATTGGAACG |
| 4 | Harpin inducing protein-like | HIN1 | Not assigned | SA, hypersensitive response and senescence | Forward: TCGTCCTCGGAGTCATTGCATTAG  Reverse: TCGAATTGTGTCAATGTGGCATCTG |
| 5 | HMG-CoA reductase | HMG | Secondary metabolism | Isoprenoids, mevalonate pathway | Forward: TGATGGCAAGGACCTCCATATTTCC  Reverse: TCTCTGTTGGCACCTTTCACTCC |
| 6 | Defensin protein precursor | PDF | Biotic stress | PR protein, defence | Forward: ATGGCTACTGAGATGGGACCAATG  Reverse: TCTCACAGACCGAGGCACAATTC |
| 7 | Glucose-6-P translocator 2 | GPT2 | Major CHO metabolism | Starch synthesis, sugar transporter | Forward: AATGGGCAGTCTCGGTGAATGAAG  Reverse: AATTGCAGCTAGGAAGGGCCAA |
| 8 | Isoflavone reductase homolog | IFR | Secondary metabolism | Phenylpropanoids, isoflavonoids | Forward : AATTCGCCGTGTTGTTGAGGCT  Reverse: AGCTCCAGGCTGTGCCAAAT |
| 9 | Pyruvate decarboxylase | PDC1 | Fermentation | Fermentation | Forward: TTGACACGTGTAAGCCACAGCA  Reverse: AGTTCAACGGAACGGCTGGATT |
| 10 | 17.7 kD class I small heat shock protein | HSP | Abiotic stress | Protein folding and stabilization | Forward : AACAGAGTGTTGAGAGTGAGCGGA  Reverse: TCCAGAACTTCCCGTAGCTCCTTT |
| 11 | Calmodulin-like protein | CML | Signalling | Calcium signalling | Forward : GTCGACTAGGTGAGTCAACCTCCATT  Reverse TCATCAAAGCTGAGGACTCCATCTCC |
| 12 | Nt-iaa4.5 deduced protein | Aux/IAA | Hormone | Auxin signalling | Forward: AGTTATTGGATGGCCACCTGTTCG  Reverse: AAATAAGCCGCACCATCCATGCTC |
| 13 | Ethylene-responsive transcriptional co-activator | ERTC | Hormone | Ethylene signalling, multiprotein bridging | Forward: TGAGGAAGAAGTTGAGGAGGAGGA  Reverse: TCACTCACCACACCCATACTCTTCT |
| 14 | Aspartic proteinase inhibitor P2B4 | API | Not assigned | Endopeptidase inhibitor activity | Forward: GTCGTGATGATGAACTTTCTTGTGCC  Forward: TTCGGTTGACAAGAAGCCAAACGC |
| 15 | WRKY-type DNA binding protein | WRKY | RNA | Regulation of transcription, defence | Forward: TGGCAAGCATGGAAACTCCAAGAG  Reverse: CTTGGCTCCTTGTTTGGAAAGCAC |
| 16 | Early nodulin 93 | ENOD | Not assigned | Putative transporter, cell wall | Forward: GCTACTGCTATTCCTACTTATGCTTG  Reverse: CAGCAACTGTGGAGACAATGAGAG |
| 17 | Spermidine synthase 1-like | SPDS | Transferase activity | Polyamine synthesis | Forward: CAACTCACAGAGCGGGATGAATG  Reverse: CCTCCTCCAATAACCAACACCTTTC |
| 18 | Patatin | PAT | Stress response | Storage protein | Forward: AGCTTTGACAGAAGTTGCCATCTC  Reverse: GGAGCTGCTGCTGTGGAATAAC |
| 19 | Trehalose 6P synthase | TPS | Minor CHO metabolism | Trehalose metabolic process | Forward: CAGGATGTAAGCAAAGGTCTGGTG  Reverse: TCGTCCGATCTGTCATCACCTATG |
| 20 | SNF1-related protein kinase regulatory subunit gamma-1 | KING1 | Carbohydrate metabolic process | Energy sensing, growth regulation | Forward: CCTGAATCTCCAACAGCGACTTCT  Reverse: GACCCTGAAATGTCCCGAACCT |
| 21 | Alanine aminotransferase 2 | AlaAT2 | Cellular amino acid metabolism | Alanine synthesis | Forward: GCAAGACGTGCGAAGACACTAGAA  Reverse: TGGGTAAGTTGATGCGTGGAAATAGA |
| 22 | Protein phosphatase 2C 63 | PP2C | Protein | Cellular protein modification process | Forward: AGTGGTCTATTGTCGAGGAGTTTGG  Reverse: CCCGCTTCAAAGGAACAGGATTTC |
| 23 | Bidirectional sugar transporter | Sweet | Carbohydrate transmembrane transport activity | Sugar transport | Forward: TGGCTCTTCGTGTTTGGAGTCTTAG  Reverse: TTTCTTTGCAGATTCGTCGGAATGC |
| 24 | Glycerol kinase, NHO1-like | GK | Glycerol metabolic process | Glycerol metabolism, non-host resistance | Forward: TGCGGTTCAGTGGCTAAGAGATAG  Reverse: CGAAATAAACTCCTCCTGTTGAGGAAAC |
| 25 | Protein TIFY 10A-like, JAZ1-like | JAZ1 | Not assigned | JA signalling repressor | Forward: TGAGAAATCTGGTGAGTCGGTTCAG  Reverse: GGTTCCGATTCAGCCTTCATTGC |
| 26 | Lipoxygenase 3 | LOX3 | Hormone | Fatty acid biosynthesis, jasmonate biosynthesis | Forward: TTGCTTTACTCCTGGTCGCTACTG  Reverse: GTTTCAGCCCATGAGGTTGTGTTG |
| 27 | CCR4 associated factor 1-like | CAF1 | Not assigned | mRNA decay, defence and stress | Forward: GCTGACGGTAACCTTCCTGATTTG  Reverse: CGATGGAGTCTGGAGCGTAGAG |
| 28 | MLP-like protein 34 | MLP34 | Not assigned | PR protein, defence | Forward: AAAGGTTAGTGACATTCAAGGCATTTG Reverse: AGCCAAGTTGTTATCTCCTTCATTCTC |
| 29 | Eukaryotic elongation factor 5A3 | EF5A3 |  | Housekeeping gene | Forward: GGAGGAGGTGGCTGAAGATTGG  Reverse: GGCTGATTGTGGTTCTGGTCTTATAC |
