# Supplementary figures and images for: Effect of wet storage conditions on potato tuber transcriptome, phytohormones and growth
Source: BMC Plant Biol. 2019 Jun 17;19:262. doi: 10.1186/s12870-019-1875-y (PMC6580497; doi:10.1186/s12870-019-1875-y)

## Verification of microarray data with qRT-PCR

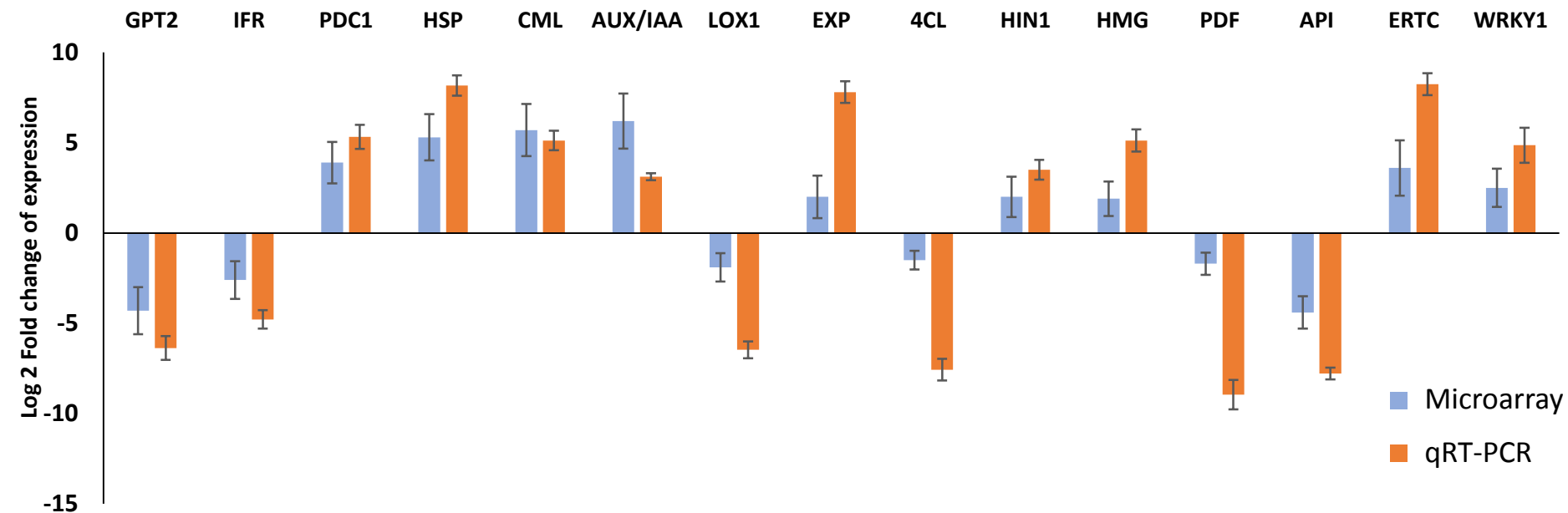

## Verification of RNA-Seq data with qRT-PCR

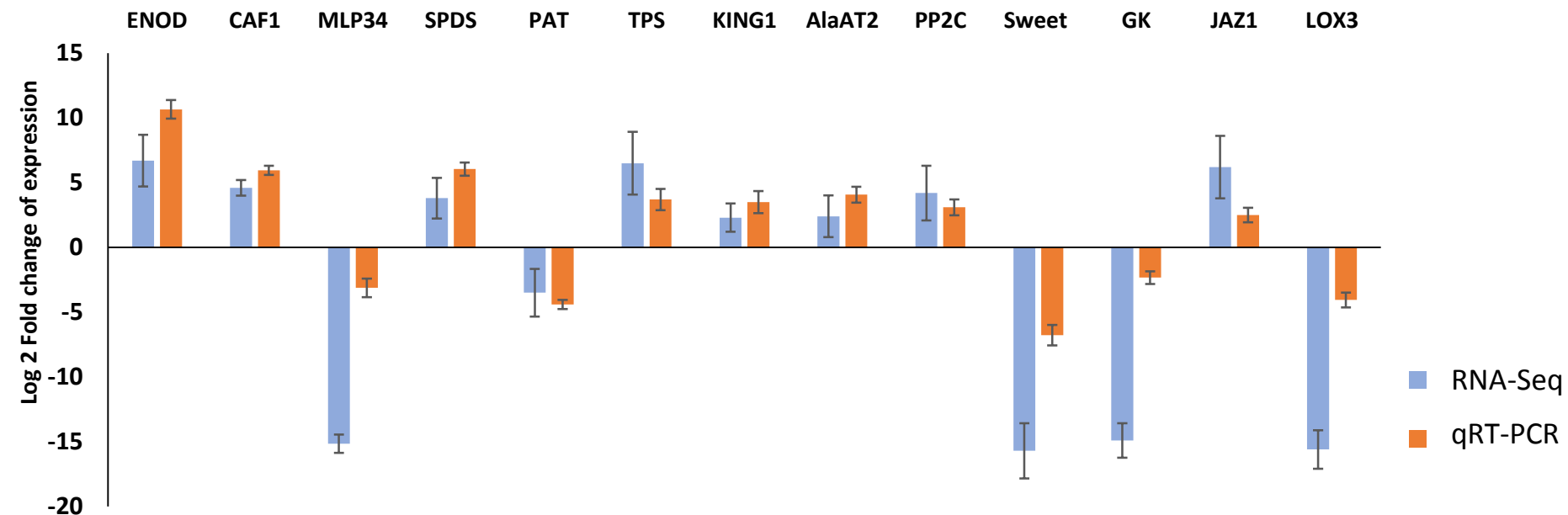

Supplement: Supplementary file 5 — Comparison of RNA-Seq and microarray analysis with qRT-PCR validation assays. Quantitative measurement of gene expression was determined with qRT-PCR for 15 DEGs in microarray (A) and 13 DEGs in RNA-Seq results (B). Data were obtained from three independent cDNA sets from three independent experiments, normalized to eukaryotic elongation factor 5A3 and expressed as the means of log2 (ΔΔCt) ± SEM (standard error of the mean). The statistical analysis for the data was performed with coefficient correlation analysis between microarray or RNA-Seq data and qRT-PCR data (log2 fold change) analyzed by the Pearson test (P < 0.05), which resulted in strong correlation between the analysis methods as indicated in Fig. 4. (PDF 180 kb) [file 12870_2019_1875_MOESM5_ESM.pdf]
